# Role of E2-Ub-conjugating enzymes during skeletal muscle atrophy

**DOI:** 10.3389/fphys.2015.00059

**Published:** 2015-03-10

**Authors:** Cecile Polge, Didier Attaix, Daniel Taillandier

**Affiliations:** UMR 1019 Nutrition Humaine, Institut National de la Recherche AgronomiqueSaint Genès Champanelle, France

**Keywords:** ubiquitin, proteasome, E2 ubiquitin conjugating enzyme, E3 ubiquitin ligase, skeletal muscle, atrophy

## Abstract

The Ubiquitin Proteasome System (UPS) is a major actor of muscle wasting during various physio-pathological situations. In the past 15 years, increasing amounts of data have depicted a picture, although incomplete, of the mechanisms implicated in myofibrillar protein degradation, from the discovery of muscle-specific E3 ligases to the identification of the signaling pathways involved. The targeting specificity of the UPS relies on the capacity of the system to first recognize and then label the proteins to be degraded with a poly-ubiquitin (Ub) chain. It is fairly assumed that the recognition of the substrate is accomplished by the numerous E3 ligases present in mammalian cells. However, most E3s do not possess any catalytic activity and E2 enzymes may be more than simple Ub-providers for E3s since they are probably important actors in the ubiquitination machinery. Surprisingly, most authors have tried to characterize E3 substrates, but the exact role of E2s in muscle protein degradation is largely unknown. A very limited number of the 35 E2s described in humans have been studied in muscle protein breakdown experiments and the vast majority of studies were only descriptive. We review here the role of E2 enzymes in skeletal muscle and the difficulties linked to their study and provide future directions for the identification of muscle E2s responsible for the ubiquitination of contractile proteins.

## Introduction

Skeletal muscle atrophy is a common adaptation of the organism during disuse (denervation, unloading) and various diseases (cancer, sepsis, diabetes, kidney failure, etc.). Compelling data demonstrated that an increased proteolysis is the main factor explaining muscle wasting (Attaix et al., 2005), and several studies suggest that the ubiquitin proteasome system (UPS) is responsible for most of this adaptation, including in humans (Polge et al., 2011). Amongst the overall myofibrillar proteins, actin and myosin heavy chains (in atrophying skeletal muscle) and troponin I (in cardiomyocytes) were confirmed to be UPS substrates (Kedar et al., 2004; Clarke et al., 2007; Fielitz et al., 2007; Heng et al., 2008; Polge et al., 2011). Other potential substrates of the UPS during muscle atrophy include other members of the troponin family, myosin light chains and telethonin (Kedar et al., 2004; Heng et al., 2008; Cohen et al., 2009).

In parallel, most efforts were put on deciphering the mechanisms by which myofibrillar proteins are targeted and degraded by the UPS. Covalent modification of proteins by ubiquitin (Ub) is a highly sophisticated and polyvalent signal as Ub linked to substrate can be monomeric, attached in chains using any of the seven internal Ub lysines or even combined with other Ub-like modifiers (Ravid and Hochstrasser, 2008; Kravtsova-Ivantsiv and Ciechanover, 2012; Ciechanover and Stanhill, 2014). Whereas several Ub modifications (e.g., K63 chains) lead to proteasome-independent degradation or non-proteolytic fate for the target (Panier and Durocher, 2009; Wertz and Dixit, 2010; Sandri, 2013), proteins carrying Ub chains linked through K48 (or sometimes K11) are *bona fide* substrates for the 26S proteasome. The latter recognizes these Ub chains as a degradation signal, trims the Ub moieties and degrades the target proteins into small peptides. The whole process is highly specific and tightly regulated in response to catabolic stimuli to avoid unwanted degradation of proteins. The first steps of the UPS are dedicated to substrate recognition and thus represent a crucial point for controlling substrate fate together with a potential entry for developing therapeutical strategies. Ubiquitination of substrates involves several hundreds of enzymes distributed in three classes that act in cascade (Polge et al., 2013). Ub is first activated by a single E1 (Ub activating enzyme) that transfers high energy Ub to one of the 35 E2s (Ub conjugating enzymes) (Van Wijk and Timmers, 2010). The E2s transfer Ub on target proteins in conjunction with the third class of enzymes, namely E3 ligases (>600, Metzger et al., 2012). An E2 is able to cooperate with different E3s and *vice versa*, which enables the specific targeting of virtually any cellular protein. E3s recognize the target protein to be degraded and thus bring specificity to the ubiquitination machinery but most E3s lack enzymatic activity so that the E2-E3 couple is functionally more relevant. Different E3 ligases have been implicated in muscle development and/or atrophy. For example, MuRF1 is a muscle-specific E3 ligase regulated in nearly any catabolic situation by different transcription mediators like FoxOs (see Bodine and Baehr, 2014 for a recent review). Interestingly, MuRF1 (and perhaps the MuRF3 isoform) is able to target the major myofibrillar proteins for subsequent degradation by the 26S proteasome (Kedar et al., 2004; Clarke et al., 2007; Fielitz et al., 2007; Polge et al., 2011). However, a yet unanswered question is about the identity of the E2s that work in pairs with MuRF1. Indeed, MuRF1 belongs to the RING finger E3 ligase family (the most numerous) that does not possess any catalytic activity and relies on E2 enzymes for targeting the proteins to be degraded. Other RING (Really Interesting New Genes) E3 ligases like Trim32 or TRAF6 may have a role in the atrophying program and also need specific E2s for correctly targeting substrates for degradation (Kudryashova et al., 2005; Hishiya et al., 2006; Kumar et al., 2012). E2 enzymes determine the type of chain built on the substrate and thus whether the ubiquitination of the target protein is dedicated to degradation or to other fates (signaling, modulation of activity, etc.). In addition, E2s bound to their cognate E3 are positioned in such a way that the residues ubiquitinated in the substrate are specifically chosen (David et al., 2010; Van Wijk and Timmers, 2010; Napolitano et al., 2011). Thus, E2s are central players in the ubiquitination machinery but the exact role of E2s in the development of skeletal muscle atrophy is still an open question.

Thirty-five E2s (plus 2 putative) are described in the human genome and have been grouped in 4 different classes (Van Wijk and Timmers, 2010). Class I only possesses the catalytic core/Ubiquitin Conjugating (Ubc fold) domain, class II and III have N- or C-terminal extensions respectively and class IV possesses both. Classification of E2s is still debated as they are grouped in 17–18 families depending on authors (Jones et al., 2002; Michelle et al., 2009). For easiest comprehension we will refer in this manuscript to the current nomenclature (UBE2x) with x defining each individual E2 enzyme. An intriguing feature is the presence of highly similar isoforms in some E2 families. For example, UBE2A and UBE2B share 96% homology at the protein level (Adegoke et al., 2002), raising an important question: do they have redundant functions? While pioneering experiments tended to globally attribute roles to the whole family, more recent data may modify this point of view. Indeed, our knowledge on the role of E2s during skeletal muscle atrophy relies almost exclusively on descriptive observations (mRNA levels) or on *in vitro* ubiquitination assays. The former are not really informative about mechanisms and specific features of E2s may bias the latter. We will discuss in this review our knowledge about E2s in skeletal muscle and focus more deeply on the two families that gather most data, including their potential link with one of the most important E3 during muscle atrophy (MuRF1). We will then address the particular features and pitfalls that have impeded to clearly depict the roles of E2 enzymes in atrophying muscles and future direction that should be developed for better deciphering UPS roles.

## UBE2 enzymes and muscle atrophy

### The role of the UBE2B family in muscle atrophy: the everlasting question?

#### Expression levels

Two class I members are present in this family, UBE2A/HR6A and UBE2B/14-kDa E2/HR6B (also referred as E2-17 kDa in humans), the latter being the most studied/tested E2 enzyme in skeletal muscle so far. These two members were among the first identified E2 enzymes, are present in different organs and share high identity both at the mRNA (80%) and protein (96%) levels in mammals (Koken et al., 1991; Adegoke et al., 2002). Although predominant in testis, pioneering work by Simon Wing's laboratory found that UBE2B is abundant in skeletal muscle and regulated upon fasting and by insulin (Wing and Banville, 1994; Wing and Bedard, 1996; Adegoke et al., 2002). One particular feature of UBE2B is that two mRNAs are present in mammals (1.2 and 1.8 kb), the smaller one being particularly sensitive to catabolic situations. Since this early work, different laboratories, including ours, found that UBE2B mRNA levels are up-regulated in nearly any catabolic situation (summarized in Table 1). These data comprise different models and muscles, from human to flies and from phasic to anti-gravity muscles. In addition, UBE2B expression is also well correlated to the expression of 26S proteasome subunits in atrophying muscles. It should be emphasized that the systematic recruitment of UBE2B is skeletal muscle-specific, as UBE2B is not modified in atrophying or hypertrophying heart (Razeghi et al., 2006). In summary, UBE2B mRNA levels are tightly linked to muscle wasting whatever the catabolic stimuli is, which suggests major roles of UBE2B downstream a ubiquitous atrophying program and that UBE2B targets are common to many catabolic states.

**Table 1:** **Variation of UBE2B mRNA levels in anabolic and catabolic situations**.

	**UBE2B**	**Conditions**	**References**
**ANABOLIC SITUATIONS**			
	↘	Insulin, IGF-1	Wing and Banville, 1994; Wing and Bedard, 1996
**CATABOLIC SITUATIONS**			
Non pathological states	↗	Fasting	Wing and Banville, 1994; Adegoke et al., 2002; Kee et al., 2003
		Vitamin D deficiency	Bhat et al., 2013
		Immobilization / unweighting	Taillandier et al., 1996; Yimlamai et al., 2005
		Aging	Combaret et al., 2005
		Burn injury	Fang et al., 2000; Chai et al., 2002
		Head trauma	Mansoor et al., 1996
Pathological and injury states	↗	Hyperthermia	Smith et al., 2005
		Mechanical ventilation	Mcclung et al., 2008
		Glucocorticoid treatment	Dardevet et al., 1995; Chrysis and Underwood, 1999
		Diabetes	Lecker et al., 1999
		Cancer	Lorite et al., 1998; Combaret et al., 2002; Khal et al., 2005; Mackenzie et al., 2005
		Sepsis	Voisin et al., 1996; Fang et al., 2000; Fischer et al., 2000
		Biliary cirrhosis	Lin et al., 2005
		Programmed cell death of skeletal muscle	Haas et al., 1995
		Increased ROS levels	Li et al., 2003

*UBE2B mRNA levels were depressed upon insulin or IGF-1 treatment*.

*UBE2B was upregulated at the mRNA level in any of the mentioned catabolic situations*.

If a proteolytic enzyme is implicated in the systematic degradation of skeletal muscle proteins in atrophying conditions, we could expect this enzyme being also down-regulated during recovery processes and/or when anabolic stimuli are delivered to skeletal muscle. Accordingly, using a non-pathological atrophying model (unweighting), we found that UBE2B was among the first enzymes down regulated after reloading of animals. Actually, UBE2B mRNA levels are severely repressed from +342% during the atrophying phase to −68% as soon as 18 h after reloading, which is much more sensitive than any of the UPS components tested (Taillandier et al., 1996, 2003). Interestingly, UBE2B mRNA is controlled by anabolic factors, as both insulin and IGF-1 lower UBE2B mRNA levels in cultured L6 myotubes but by a different mechanism. While insulin lowers UBE2B mRNAs by playing on transcription, IGF-1 has a direct effect on UBE2B mRNA stability and increases its degradation (Wing and Banville, 1994; Wing and Bedard, 1996). The impact of IGF-1 on UBE2B mRNA was also observed in rats treated with dexamethasone (Dex) and in septic animals (Dardevet et al., 1995; Chrysis and Underwood, 1999; Fang et al., 2000). However, depressing UBE2B mRNA and other components of the UPS had only a limited impact on muscle mass when catabolic stimuli were present and this absence of a clear effect might reflect compensatory mechanisms, e.g., an adjustment of the protein synthesis/degradation balance. Muscle mass is submitted to daily variations due to the alternation of postabsorptive (catabolic) and postprandial (anabolic) phases, which is a physiological mechanism regulating protein homeostasis. Aging is characterized by the loss of the postprandial inhibition of proteolysis, in which different components of the UPS are normally upregulated, including UBE2B. Interestingly, leucine supplementation restores the postprandial inhibition of proteolysis by decreasing UBE2B and other UPS mRNA levels (Combaret et al., 2005). *In vitro* experiments using C2C12 myotubes suggested that the leucine effect was mediated by insulin (Sadiq et al., 2007).

Expression at the mRNA levels may not be sufficient for proving that UBE2B is important for muscle homeostasis. However, few studies confirmed a role for UBE2B at the protein level partly because most antibodies cross-react with the isoform UBE2A. In rats submitted to unweighting atrophy, we found that increased UBE2B mRNA levels correlated with efficient translation (Taillandier et al., 1996). Using a moderate catabolic model (fasting), other authors found that UBE2B protein levels were not modified while mRNA levels were elevated (Adegoke et al., 2002). These authors concluded that increased mRNA levels were probably required for maintaining enzyme levels, which is suggestive of a posttranscriptional regulation. The simplest explanation is that UBE2B turnover is dramatically increased in atrophying muscle even though this remains to be established.

#### Ubiquitination activity: E3 partners and substrates

Different UBE2B ubiquitinating activities were first observed using *in vitro* experiments. Monoubiquitination of histones was reported but also polyubiquitination and subsequent degradation of α-lactalbumin (Sung et al., 1991; Wing et al., 1992). UBE2B-dependent ubiquitination was mainly performed using purified Ubr1/E3α or cell extracts enriched in E3 ligase activity. Like other E2 enzymes, the cellular context greatly influence the type of ubiquitination so that we will mainly focus on the ubiquination assays performed in skeletal muscle.

Using non-catabolic skeletal muscle extracts, Alfred Goldberg's laboratory found that UBE2B was a major actor of the so-called N-end-rule pathway, i.e., the labeling and subsequent degradation of proteins dependent on the N-terminal amino acid of the degraded protein (Solomon et al., 1998a). Accordingly, UBE2B and its isoform UBE2A were reported to represent nearly half of total ubiquitin conjugation in skeletal muscle and other cell types, underscoring the importance of the UBE2B family (Rajapurohitam et al., 2002; Siepmann et al., 2003). Other studies used muscle cell extracts from catabolic animals (diabetes, cancer, and sepsis) and found that the N-end rule pathway (that includes the enzymes UBE2B and Ubr1 mostly explained increased ubiquitination rates (Solomon et al., 1998a; Lecker et al., 1999). The use of a dominant negative UBE2B and inhibitors of Ubr1 confirmed this hypothesis and suggested that 60–80% of the ATP-dependent degradation of soluble muscle proteins was mediated by the N-end rule pathway (Solomon et al., 1998b). Accordingly, hypoactivation of the UPS and low polyUb conjugate levels in skeletal muscles were attributed to a repression of the N-end rule pathway in thyroidectomized and hypophysectomized rats (Solomon et al., 1998a). A tricky observation was that UBE2B ubiquitinated model substrates like lysozyme or α-lactalbumin but not the main contractile proteins actin and myosin (Solomon et al., 1998b; Lecker et al., 1999) and that the impact of the N-end rule pathway was only demonstrated in muscular soluble extracts. However, different explanations may relativize this absence of effect of UBE2B on the main contractile proteins. First, the fact that UBE2B (and more globally the N-end rule pathway) acts mainly on the soluble fraction from skeletal muscle extracts does not exclude a role on contractile protein turnover. Indeed, a fraction of these proteins are always present in such cell extracts and may represent a transient rapidly evolving pool (Neti et al., 2009). Second, a single E3 was used in ubiquitination assays and we know that E2s work with several partners in cells. Finally, another aspect is the potential redundancy between UBE2A and UBE2B but this point will be addressed in the last paragraph of this review. It seems very unlikely that a so abundant E2 (UBE2B) would be restricted to a single E3 in skeletal muscles since in other organs/cell types UBE2B interacts with the E3 ligases Rad18, Ubr2, Bre1, and Mdm2 for controlling histones, p53, PCNA and myc proteins ubiquitination respectively (Gross-Mesilaty et al., 1998; Kim et al., 2009; An et al., 2010; Hibbert et al., 2011; Chen et al., 2012). Depending on the cognate E3, UBE2B is implicated in different metabolic pathways from transcriptional silencing during spermatogenesis (An et al., 2010) to DNA repair (Hibbert et al., 2011). In skeletal muscle most studies focused on Ubr1-UBE2B but the list of cognate E3s and the roles of each UBE2B-E3 couples are probably far from being closed.

An important point when addressing the biochemical role of E2 enzymes is to determine the kind of ubiquitin linkages promoted. However, no study reported the type of Ub chain(s) UBE2B promotes in skeletal muscle and we can only elaborate hypotheses based on studies performed in other cell types. Like other E2s, UBE2B is able to build different Ub chains depending on the context and the ligase. *In vitro* assays performed without any E3 ligase showed that UBE2B is able to promote K11, K48, and K63 Ub chains (David et al., 2010). As underlined by the authors, this kind of assay only shows the (minimum?) capacity of E2 enzymes for building Ub chains. This was confirmed for UBE2B, as pioneering studies showed that N-end rule substrates were labeled with K48 chains when Ubr1 was used as an E3 ligase (Chau et al., 1989). Likewise, UBE2B-dependent ubiquitination of β-catenin was observed in breast tumor cells, which was attributed to K63 Ub chain formation (Shekhar et al., 2008). In the latter case, UBE2B did not promote protein degradation by the 26S proteasome but rather enabled β-catenin to escape degradation. K11 Ub chains were observed *in vitro* but clear data about the implication of UBE2B for building these chains *in vivo* are still lacking (Hibbert et al., 2011). PolyUb chain formation is not the only signal UBE2B can promote, as its activity is restricted to monoubiquitination of PCNA and histone H2B when combined with the E3 ligases Rad18 and Bre1 respectively (Kim et al., 2009; Hibbert et al., 2011).

In conclusion, Ub chain formation and the substrates targeted by UBE2B in skeletal muscle are still a mystery and we still do not know whether UBE2B is able to target myofibrillar proteins.

### The UBE2D family: the false friends?

#### Expression levels

The UBE2D/UBC4/UBC5/17 kDa-E2/UbcH5 family belongs to class I E2 enzymes like UBE2B (Van Wijk and Timmers, 2010). Like the UBE2B family, the UBE2D isoforms share high protein homology ranging between 90 and 93% identity in humans and most studies considered that they share common properties. This is the second most studied family in skeletal muscle as UBE2D1 (UbcH5a), D2 (UbcH5b), and D3 (UbcH5c) are commonly used for their ability to build Ub conjugates on substrates *in vitro* with MuRF1 as an E3 ligase (Kedar et al., 2004; Clarke et al., 2007; Fielitz et al., 2007; Polge et al., 2011). Indeed, *in vitro* assays using UBE2Ds and MuRF1 allowed the polyubiquitination of the main contractile proteins like actin, myosin heavy chain and troponin. As MuRF1 is the major E3 ligase that targets myofibrillar proteins for their degradation by the 26S proteasome, a tempting shortcut is to associate UBE2D enzymes and muscle atrophy. However, this might not be so simple.

In rats submitted to Dex treatment, UBE2D2 mRNA levels were enhanced together with other UPS components. By contrast with the latter, UBE2D2 was insensitive to IGF-1 treatment (Chrysis and Underwood, 1999). This lack of effect of anabolic stimuli may be due to switches from different group of targets using different E3 ligases but this remains to be clarified. The most complete study about UBE2D was performed by Alfred Goldberg's laboratory that checked in rodents' muscles the expression levels of proteolytic genes in different catabolic situations, i.e., fasting, diabetes, chronic renal failure and cancer (Lecker et al., 2004). UBE2D2 was chosen as representative of the family, but was not up-regulated in any of the catabolic conditions tested. This suggests that glucocorticoid treatment is one of the few if not unique catabolic situation that induces an overexpression of UBE2D2. Other observations do not favor a role of UBE2D in the muscle atrophying program (see below) and thus caution should be taken when associating MuRF1 and UBE2D for identifying substrate targeting mechanisms in skeletal muscle.

#### Ubiquitination activity: E3 partners and substrates

UBE2Ds are very promiscuous enzymes that exhibit ubiquitination activity with a huge number of E3 ubiquitin ligases toward various substrates. A good example of this wide interaction pattern was provided by E2-E3 interaction screenings. Using 10 different E3 ligases (including the MuRF family: MuRF1, MuRF2 and MuRF3) and 11 different E2 enzymes, only the UBE2D family (UBE2D2 and D3) was able to promote *in vitro* ubiquitination with all the E3 ligases tested (Marblestone et al., 2013). Similarly, only the UBE2D members tested (UBE2D1, D2, and D3) were able to ubiquitinate myosin heavy chain *in vitro* with MuRF1 or MuRF3 as cognate E3 ligases (Fielitz et al., 2007). Large screening using Yeast two-Hybrid (Y2H) assays lowered the percentage of positive interactions between UBE2D and E3 ligases but this family still remained in the top of E2-E3 interactions as they totalized 34–40% of all the detected interactions (Markson et al., 2009; Van Wijk et al., 2009; Napolitano et al., 2011). Unfortunately, the MuRF family was omitted in these wide screenings.

UBE2D enzymes lack specificity for chain linkage *in vitro*, although they exhibit a preference for building K11, K48, and K63 chains (Kim et al., 2007; Ye and Rape, 2009). UBE2Ds generally also exhibit low specificity toward E3 enzymes and substrates when used *in vitro* (Brzovic et al., 2006; Wenzel et al., 2011b). These authors suggested that this due to peculiar Ub binding on the backside region of UBE2D. It is believed that the backside face of UBE2 enzymes opposite to the catalytic pocket does not participate to the covalent Ub attachment to the substrate. In fact, the use of the backside region extends to ubiquitin-like modifications as UBE2I, the SUMO-specific E2, binds SUMO through the backside region for promoting sumoylation (Knipscheer et al., 2007). Ub binding through the backside of UBE2D (and some other E2s) ended up with a proposed mechanism in which Ub bound to the back face of UBE2D elicits an aggregation of UBE2D enzymes that promotes a highly efficient processive ubiquitination mechanism (Ye and Rape, 2009). Such an aggregation of UBE2D enzyme may be responsible for the high efficacy of UBE2D2 within *in vitro* ubiquitination assays whatever the E3 and the substrate. If true, this raises the question of the physiological relevance of MuRF1-UBE2D ubiquitination assays, not for the recognition of the substrate that belongs to MuRF1 but for the kind of chains built on substrates and the role of UBE2D enzymes in atrophying skeletal muscle. Altogether, the UBE2D family is far from being a pretender for playing an important role during skeletal muscle atrophy in view of the available data.

### Other UBE2s

Few other studies addressed the remaining E2 enzymes in atrophying skeletal muscles. A single study screened for several E2 enzymes and found that UBE2G1 was up-regulated in skeletal muscles from rodents undergoing chronic renal failure (CRF), diabetes, fasting, and cancer (Lecker et al., 2004). The second isoform of the family (UBE2G2) was also up-regulated in CRF and cancer animals while UBE2L3 and UBE2O were enhanced only during fasting. UBE2L3 was also up-regulated during immobilization in pigs (Banduseela et al., 2009) and denervation induced an increased expression of UBE2O in the gastrocnemius muscle from mice (Gomes et al., 2012). It should be noticed that UBE2O was formerly depicted as predominantly expressed in skeletal muscle and heart (Yokota et al., 2001), but this was due to erroneously loaded commercial blots as this enzyme is highly expressed in other organs. Indeed, UBE2O is abundant in bones (NextBio body Atlas https://www.nextbio.com) and plays an important role in osteoblast formation (Hao et al., 2013; Zhang et al., 2013). In fact, UBE2O overexpression in C2C12 skeletal muscle cells induces an osteoblastic program and this E2 is thus a poor candidate for playing a role during skeletal muscle atrophy. UBE2H is implicated in the negative control of the IGF-1 and insulin-signaling pathway with the E3 ligase MG53/Trim72 during myogenesis in cultured C2C12 cells and in mice (Yi et al., 2013; Nguyen et al., 2014b). However, the implication of UBE2H in atrophying conditions has not been reported yet. UBE2J1 was up-regulated in chronic renal failure (CRF), diabetes, fasting, cancer and immobilization (Lecker et al., 2004; Banduseela et al., 2009), suggesting a recurrent role of this E2 in the atrophying process. However, UBE2J1 (and its isoform UBE2J2) is a membrane protein present in the endoplasmic reticulum (ER) (Oh et al., 2006), which thus makes it a poor candidate for the targeting of the contractile apparatus. Indeed, this E2 enzyme is a tail-anchored protein facing the cytoplasm and is probably involved in the ubiquitination of misfolded or misassembled proteins. Finally, UBE2V2 was up-regulated during spaceflight (Allen et al., 2009).

In summary, very few studies attempted to address the role of E2 enzymes in skeletal muscle during atrophying processes. A clear picture of the E2s up-regulated in different catabolic conditions would be a first step. It is clear that most E2-E3 interactions are weak and/or transient, which renders more difficult the detection of E2-E3 couples. However, Y2H, SPR or Fluorescence Resonance Energy Transfer (FRET) technologies should prove to be useful tools (and already proved to be in non-muscle cells) for tracking relevant interactions. Using knockdown and overexpression approaches should also be valuable for addressing the physiological targets of E2 enzymes. The picture at that time is kind of a black box and we do not know which E2(s) is(are) important/implicated in skeletal muscle atrophy, which E3 enzymes are working with them and what are their respective targets.

## Specific features of E2 enzymes and pitfalls

Information about ubiquitination processes come from structures of E2-E3 couples identified in non-muscle cells but these data are highly valuable for future studies addressing ubiquitination specificity and role in the atrophying skeletal muscle. Dissecting all the knowledge about E2-E3 structures is beyond the scope of this review and readers are redirected to excellent reviews (Ye and Rape, 2009; Van Wijk and Timmers, 2010; Wenzel et al., 2011b). We will discuss the main features that should be taken into account when studying ubiquitination in skeletal muscle.

### Structure and ubiquitination specificity

With the exception of UBE2O (230 kDa), E2 enzymes are relatively small proteins, as class I E2s possessing only the catalytic core (or Ubc fold) are within the 14–20 kDa range. Even E2 enzymes harboring N and/or C-terminal extension like UBE2U barely exceed 35 kDa. The Ubc fold has a globular shape and concentrates all the necessary interaction surfaces for achieving Ub conjugation (Van Wijk and Timmers, 2010; Wenzel et al., 2011b). Indeed, and despite their small sizes, each E2 interacts with various proteins, and interacting partners are not restricted to E1, E3 ligases and ubiquitin. Indeed, a wide Y2H screen found that less than one third of the interactions with E2 enzymes belong to E3 ligases, which means other partners may be part of the E2 network (Markson et al., 2009). The multitude of interactions means that E2 enzymes have developed a highly rationalized structure that uses a limited number of residues for achieving a functional role (Van Wijk and Timmers, 2010; Wenzel et al., 2011b). In addition, the small number of E2 enzymes toward the vast number of E3s has pushed cells to develop highly specialized overlapping interaction domains so that each E2 interacts with a panel of E3 ligases. However, other E2s like UBE2K lack a single Phe residue crucial for E2 interaction with HECT-E3s (Homologs to the E6-AP Carboxyl Terminus E3) thus suggesting a specialization of some E2s (Huang et al., 1999). This model well illustrates the importance of a single amino acid residue in E2 roles, as the introduction of the crucial Phe in UBE2K allows the partial recovery of a UBE2D mutant that lacked E2-HECT function (Nuber and Scheffner, 1999).

**Figure 1:**
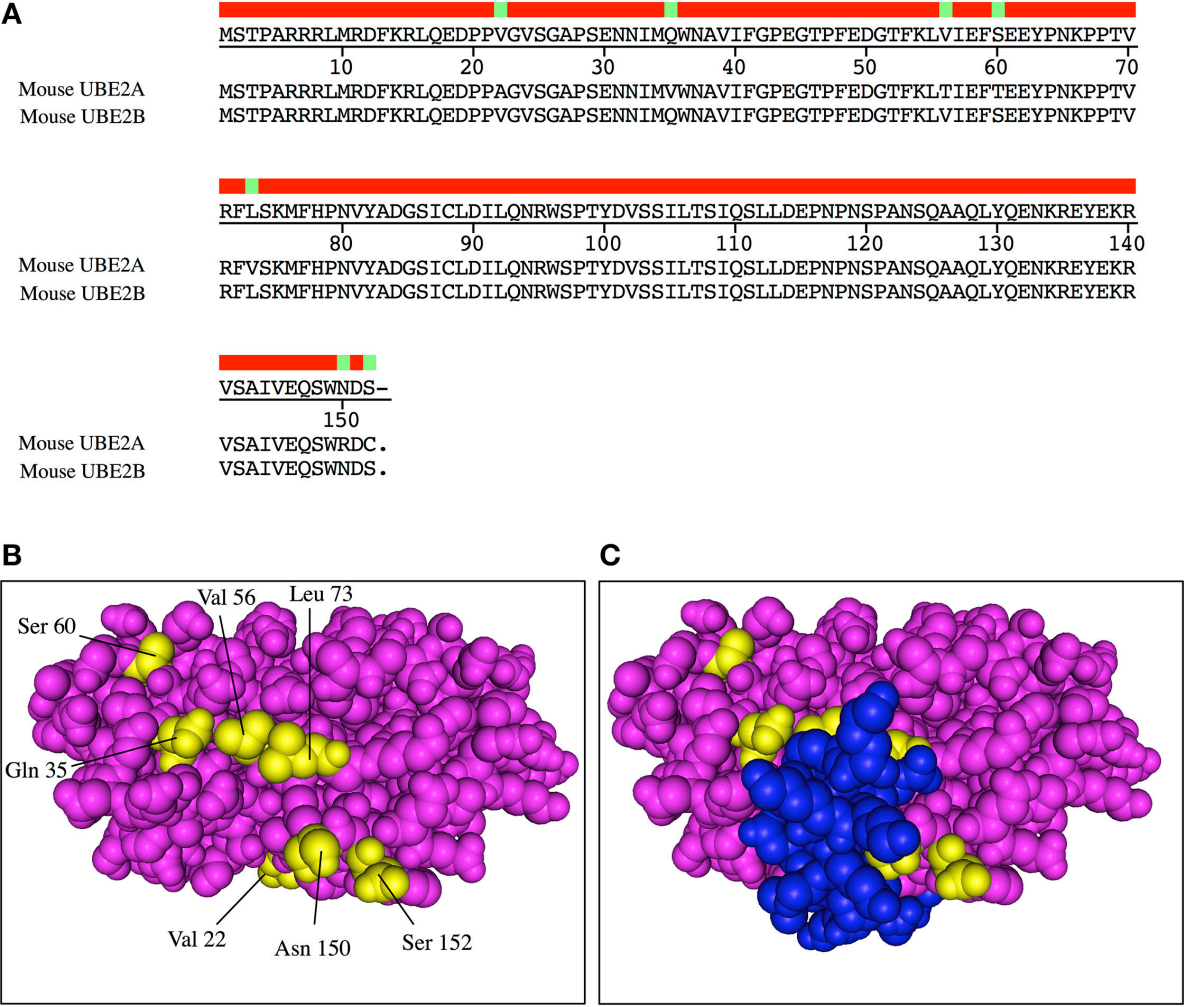
**Crystallographic structure of human UBE2B. (A)** Alignment of mammalian UBE2A and UBE2B. Consensus sequence (red bar) is shown at the top and differential residues are highlighted in green. **(B)** UBE2B structure backside view (PDB code 2YBF). Specific residues within UBE2B are shown in yellow. **(C)** The E2 binding domain of the E3 ligase Rad18 (shown in blue) covers the central area of UBE2B backside, including some of the specific residues (yellow) that are different between UBE2B and UBE2A.

Peculiar features of E2 interaction surfaces include overlapping binding sites for E1 and E3s and a limited number of crucial residues involved for each individual interaction. Three E2 regions are mainly involved in E1/E3 interactions namely the helix 1 (H1), and loops 4 and 7 (L4 and L7). The solved E2-E3 structures showed that a limited number of residues present in H1 and one or two residues in each loop are important for E2-E3 interactions (Wenzel et al., 2011b). This means that a modest number of residues govern the choice of the E3 and that small modifications on E2s modify the specificity toward E3s. For example a single mutation of Ala^96^ to Asp disrupts BRCA1-UBE2D3 interaction (Christensen et al., 2007). In addition, crucial residues for a given E2 family can be unimportant for another one, which means we can hardly predict interaction features for E2-E3 couples with unsolved structures. In addition to these canonical binding regions, the E2 backside seems also to play an important role for some E2-E3 interactions, e.g., UBE2G2 and the E3 ligase gp78 (Das et al., 2009; Randles and Walters, 2012). The G2BR domain of gp78 specifically binds the backside region of UBE2G2 with high affinity only when the E2 is charged with Ub, while it exhibits low affinity toward the uncharged UBE2G2. This mechanism is at the basis of processive ubiquitination. Clearly, some E3 ligases can interact with the backside region of E2 enzymes (see UBE2B-Rad18 structure, Figure 1) by using non-RING domains, which either enhances or inhibits processive Ub chain formation (Bailly et al., 1997; Brzovic et al., 2006; Chen et al., 2006; Notenboom et al., 2007; Das et al., 2009; Hibbert et al., 2011; Metzger et al., 2013; Nguyen et al., 2014a). For example, depending on the E2-E3 couples, interaction of E3 to the backside either favors monoubiquitination (UBE2B-Rad18, UBE2E3-AO7) or polyubiquitination (UBE2G2-gp78) underscoring the variety of modulation E2-E3 couples have developed for achieving Ub chain formation. As the backside region is probably a multifunctional binding surface, future studies may uncover other interactors/binding residues that may be related to different physiological functions.

Some E2 enzymes like the UBE2D family have the ability to build Ub conjugates *in vitro* without the help of E3 ligase, but E3 ligases are clearly needed for the orientation of the substrate and serves as scaffolds for positioning correctly the E2 and the target for optimal Ub transfer on the dedicated lysine residue (Huang et al., 1999; Zheng et al., 2000). Correct positioning is not the only reason for cells to develop a “quality control” mechanism using E3 ligases. Indeed, E2-Ub reactive species may be deleterious for cellular metabolism. A common feature for ubiquitination is the conformation modification sustained by E3 ligases. E2-Ub possess a flexible structure in which each protein is relatively independent while the RING finger of E3 ligases imposes a close structure that carefully orients Ub for achieving a highly specific ubiquitination of defined residues with the desired Ub chain (Dou et al., 2012; Pruneda et al., 2012; Soss et al., 2013; Berndsen and Wolberger, 2014). UBE2V-UBE2N heterodimer developed self-control ubiquitination mode and correctly orientates K63 for Ub chain formation onto the substrate. Indeed, UBE2V lacks catalytic activity but positions Ub between the V-N heterodimer, which brings Ub in front of the RING and allows ubiquitination by UBE2N. However, the presence of a minimal RING domain is necessary for optimal activity (Eddins et al., 2006; Yin et al., 2009). Another way for cells to control the ubiquitination process is to use a single E2 enzyme that lacks reactivity against lysine, i.e., it can not build chains even though it is able to interact with RING finger E3 ligases. UBE2L3 is believed to lack intrinsic Ub chain building ability and works primarily with HECT E3 or with RING-between-RING (RBR) ligases (Wenzel et al., 2011a). In another example of controlled ubiquitination by an E3 ligase, the role UBE2E3 alone was largely restricted to monoubiquitination while the presence of its cognate RING finger E3 ligase RNF25 greatly increased polyUb formation, thereby limiting E2 ubiquitination activity in absence of an E3 (Nguyen et al., 2014a).

The catalytic cysteine and the surrounding HPN residues form the active site of E2 enzymes but are not always sufficient to explain the ubiquitination mechanism on protein substrates. For example, UBE2G2 possesses acidic residues in flexible loops completing the HPN residues and the binding of Ub or E3 onto UBE2G2 seems a prerequisite for a more ordered structure and thus for efficient ubiquitination activity (Ju et al., 2010). This means that Ub or one of the cognate E3 might be necessary for detecting Ub conjugation *in vitro*. It is tempting to extrapolate that other E2 enzymes need E2-Ub, specific E2-E3 configuration or a third-party protein for correctly ordering the catalytic site. If true, this may explain why so few E2-E3 couples are active *in vitro* (Wenzel et al., 2011b). For UBE2Ds, the absence of such acidic residues favors constitutive ubiquitination activity, which could explain the remarkable *in vitro* activity of these E2s (Kim et al., 2007; Marblestone et al., 2013).

The role of the N or C-terminal extensions present in some E2 enzymes is far from being understood. The UBE2J family uses the C-terminal extension for anchoring to the ER membrane, which specializes UBE2J1 and J2 to ER-linked ubiquitination activity (Oh et al., 2006; Van De Weijer et al., 2014). UBE2C N-terminal extension plays a role in the ubiquitination mechanism both at the ubiquitination and target lysine levels (Summers et al., 2008) while UBE2R2 C-terminal extension may be implicated in both ubiquitination and localization (Sadowski et al., 2007). The large UBE2O is *per se* a combined E2-E3 entity (Berleth and Pickart, 1996) but it is also able to work with the E3 MAGE-L2/TRIM27, suggesting multi-functional roles for this E2 (Hao et al., 2013). More than half of the E2 enzymes exhibit extensions and future studies will have to clearly identify their roles, which may encompass localization, recognition of substrates, binding with specific E3s, modulation of activity, etc.

The above examples highlight that the Ubc fold of E2 is a compact economical structure that optimizes each contact surface. A single amino acid modification, the presence of a substrate/co-factor/specific E3 can greatly modify the E2 activity. This underscores huge constraints not always compatible with *in vitro* assays. Not all surfaces and crucial residues have been discovered yet and future studies will probably highlight new interaction with yet unknown partners (as suggested by Y2H screens) that will help for better defining E2 roles.

### E2 expression levels

Some E3 ligases exhibit a restricted expression; this is the case for the MuRF family and MAFbx whose expression is limited to skeletal, cardiac, and smooth muscles (Bodine et al., 2001; Bdolah et al., 2007; Yoshida et al., 2010; Bodine and Baehr, 2014). For E2 enzymes, the relatively small number of enzymes makes it almost impossible that a given E2 could be restricted to a single organ. However, E2 enzymes can be highly present or not detectable, which means that some restriction of expression exists and depends of the cell type/organ studied (Uhleìn et al., 2015). Data base like NextBio body Atlas (https://www.nextbio.com) may give a general picture of the expression levels of E2 enzymes. For example, only 26 E2 enzymes were detected in NIH3T3 cells both at the mRNA and protein levels (Schwanhausser et al., 2011, 2013). Similarly, important discrepancies in E2 abundance were detected between E2 enzymes in both Hela cells with ratio >200 for UBE2L3/UBE2T for example (Schwanhausser et al., 2011, 2013). Similarly, other authors found 50 times more UBE2B than UBE2D in the rat gastrocnemius muscle while similar levels of these enzymes were observed in the brain (Rajapurohitam et al., 2002). Interestingly, the much higher proportion of UBE2B over UBE2D in skeletal muscle reflected a very low abundance of UBE2D. In skeletal muscle, UBE2A and UBE2B represent a fair amount of total ubiquitination but other E2s like UBE2E1, G1, G2, H, J1, J2, L3, N, O, V1, V2, and Z are also well represented in this tissue. By contrast, others like UBE2C, K, Q1, and Q2 are quite absent or present at low levels, which brings the question of the heterogeneity of the tissue. Indeed, UBE2K is highly abundant in peripheral blood cells and the low levels of UBE2K detected in skeletal muscle may reflect the presence of multiple cell types in the tissue rather than “true” presence of UBE2K in skeletal muscle cells. Using cell culture may be an alternative but keeping in mind that myotubes are not fully differentiated muscles. Importantly, the presence of an E2 enzyme in the organ studied is a first step when studying E2-E3 interactions *in vitro* for being sure that the enzymes have a chance to interact *in vivo*.

Unfortunately, few studies have addressed the expression levels of E2 enzymes in the different tissues/organs, including skeletal muscle. However, we can hypothesize that the observed variations are probably correlated with the presence of cognate E3s, the substrates and ultimately the physiological roles of E2 enzymes.

### Interaction with substrates

When considering the Ubc fold, contacts between the E2 enzymes and the substrates they ubiquitinate is limited to the strict minimum. This is a peculiar mode of action due to the scaffold role of E3 enzymes. This means that even though E2 enzymes generally retain the catalytic role during the ubiquitination process they do not have any intrinsic affinity for their target. However, E2 extensions may play substrate-binding role and complete the pleiotropic capacity of E2 enzymes. This is the case for UBE2C, one of the Anaphase Promoting Complex (APC) E2 enzymes, that combines E2-E3 capacities thanks to its N-terminal extension (Summers et al., 2008). However, this is more an exception than the rule. Therefore, classical approaches like pulldown assays are useless for identifying the physiological targets of E2s, thus increasing the difficulty for such identifications. In addition, E2-E3 interactions are *per se* generally weak and transient, which urge the development of new approaches for elucidating the physiological role of each E2 enzyme. In a recent work, fused E2-E3 constructions were used for identifying new targets of the Mulan E3 ligase (Ambivero et al., 2014). The authors first identified Mulan E2 interactors using Y2H assays and then fused the RING-finger domain of Mulan to the identified E2s. The Mulan RING-finger-E2 fusion proteins were then used as bait for identifying new Mulan targets. This interesting approach may be used with other fusion strategies like E3-substrate to identify specific E2s.

### Posttranslational modifications

Metabolic pathways are widely regulated through posttranslational modifications, ubiquitination being one of them. Phosphorylation is commonly used for modulating enzyme activity and E2 enzyme activity can also be modulated by phosphorylation. Few studies have addressed this point but a good example comes from UBE2B-UBE2A phosphorylation (Sarcevic et al., 2002; Kumar et al., 2010). Phosphorylation of a single residue of UBE2B (Ser^120^) was shown to control the activity of this E2 enzyme. Indeed, the unphosphorylated form of UBE2B drives K48 polyUb targeting of model N-end rule substrates using the E3 Ubr1, whereas a phospho-mimic UBE2B was restricted to mono-ubiquitination (Kumar et al., 2010). This was confirmed by the abolition of histone mono-ubiquitination in Ser^120^-mutated UBE2A (Sarcevic et al., 2002). Interestingly, the Ser^120^ residue is positioned close to the catalytic Cys and Ser120Ala mutation abolished UBE2B activity, suggesting that this residue has a pivotal role for ubiquitination. This mechanism is not common to all E2 enzymes, as the paralogous residue in UBE2D exhibits no importance for ubiquitination activity (Kumar et al., 2010). Based on protein sequence, the authors predicted potential phosphorylation sites in the Ubc fold of UBE2C, UBE2G1, and UBE2R1. Phosphorylation was also observed in the acidic tail of UBE2R1 and increased ubiquitination activity followed by accelerated cell cycle in yeast (Sadowski et al., 2007; Coccetti et al., 2008). Phosphorylation-linked ubiquitination control was also suggested for UBE2J1. However, phosphorylation state did not affect stability, localization or E3 binding with the cognate E3 Parkin (Oh et al., 2006).

We can hypothesize that other E2 enzymes may be modified by phosphorylation (or other posttranslational modifications) and thus diversify the ubiquitination roles of these enzymes. More generally, if posttranslational modifications prove to be more common within E2s, this will virtually expand the number of E2 enzymes present in cells.

### *In vitro* ubiquitination assays: interpretation and usefulness

*In vitro* ubiquitination assays are widely used in the UPS world and they proved to be useful for deciphering the mechanism of ubiquitination, e.g., for determining the residues in E2 and E3 enzymes that are crucial for sustaining an efficient catalytic activity. They have been also widely used for suggesting that a target protein was the physiological substrate of an E3 ligase. However, ubiquitination assays usefulness is questionable for identifying the right E2 enzyme. A typical case is UBE2D, a promiscuous E2 enzyme that exhibits an incredible ability for Ub conjugation *in vitro*. This is detrimental as it is almost impossible to discern physiologically relevant and non-relevant activity. As discussed before, UBE2D builds *in vitro* different types of Ub chains on muscle-specific proteins in association with MuRF1 or MuRF3 (Kedar et al., 2004; Clarke et al., 2007; Fielitz et al., 2007; Polge et al., 2011). This brings the question of specificity since the type of Ub chains define the fate of the substrate, and it is astonishing that cells can deal with an E2 not able to bring specificity (Kim et al., 2007; Ye and Rape, 2009). Combined with our data, the simplest explanation is that the former ligases are not *in vivo* partners of UBE2D and that the intrinsic capacity of UBE2D to non-specifically form Ub conjugates *in vitro* was the main determinant. In addition, UBE2D may be involved in a more complicated way in the ubiquitination process. It has been shown that E2s can work in concert, which means that cooperation between at least two different E2 enzymes influences the ubiquitination process. This is the case for UBE2W and UBE2E2, this dimer initiating Ub chain initiation when combined with the E3 ligase BRCA1 and UBE2N-UBE2V1 dimer elongating Ub chains (Christensen et al., 2007). Similarly, UBE2D was proposed to only initiate ubiquitination and other E2 enzymes might be responsible for the development of Ub chains, which is the case for UBE2D-UBE2K ubiquitination activity within the APC complex (Rodrigo-Brenni and Morgan, 2007; Ye and Rape, 2009). E2 combinations represent thus a possibility of control of Ub chain formation by cells, together with posttranslational modifications and binding with other partners. These parameters are often difficult to take into account *in vitro* and, as a first step, more sensitive approaches should be favored and combined when addressing E2-E3 interactions.

### E2-E3 interaction screening

Few E2-E3 interactions are detectable using classical pulldown assays, with some exceptions like UBE2B with the E3 ligase Rad18. The reason for such transient interaction is adaptive and allows highly processive ubiquitination of a substrate. This is well illustrated by the ubiquitination process of UBE2G2 and the RING E3 gp78 (Das et al., 2013). Binding of UBE2G2 to the dedicated domain G2BR on gp78 increases UBE2G2-RING binding affinity and promotes ubiquitination of the substrate. Concurrently, Ub discharge lowers the UBE2G2 affinity for G2BR and UBE2G2 is released rapidly for being used in the next round of ubiquitination. This example highlights also the potential role of Ub charging on E2 for achieving efficient binding on the cognate E3. Similarly, when they are charged with Ub some E2 enzymes exhibit enhanced affinity for co-factors such as Ub Binding Domains (UBD) or membrane receptors (Hoeller et al., 2007; Ravid and Hochstrasser, 2007). This is the case for the yeast homolog of UBE2G2 that binds to Cue1 at the ER membrane, which localizes both ubiquitination activity and specificity toward the target proteins. Thus, while most available data suggest that E2-E3 interaction is generally stand-alone, this might not be a ubiquitous feature. Depending on the E2-E3 couples studied, the presence of binding partners and Ub may influence the affinity for a given E2 to cognate E3 ligases. This may partly explain why Y2H screens failed to find any E2 for some E3s and *vice versa* (Markson et al., 2009; Napolitano et al., 2011) and also why the two-third of the interactions observed for E2s in Y2H assays were not belonging to E3s (Markson et al., 2009). This aspect should also be taken into account in future experiments.

### Does family means redundant functions?

Some families of E2 enzymes exhibit highly similar isoforms. This is the case for UBE2A and UBE2B that share 96% identity at the protein level (Figure 1A), the UBE2D family (90–93%), UBE2E (80–85%), UBE2Q (72%), UBE2R (79%), and UBE2V (88%). These close isoforms are often considered as possessing redundant functional activities. In skeletal muscle, UBE2B knockout was inefficient for improving muscle mass in starved animals and the authors concluded that UBE2A activity was sufficient to compensate for UBE2B loss (Adegoke et al., 2002). By contrast, the very same E2 enzymes exhibited clear differential role in testis as UBE2B knockout induced male infertility (Roest et al., 1996). The reasons for such discrepancies are not clear but knockout often induces differential compensation phenomena due to long-term absence of a protein during all the development of the animal. An alternative explanation may also be that the E3 “arsenal” is different between testis and skeletal muscle. A first clue is the highly conserved sequence for each enzyme among species. There is no amino acid difference for mammalian UBE2B and only a conservative Val/Ile modification between human and Chinese turtle for example. As discussed above, a single amino acid modification within the Ubc fold can change the affinity toward E3s and the ubiquitination activity, suggesting that the seven different amino acids between UBE2A and UBE2B reflect functional differences. UBE2B structure gives another indication as six out of the seven residues form two patches located in the backside of UBE2B (Figure 1B). This region possesses at least two potential functions, non-covalent binding of Ub and interaction with E3 ligases. These residues being solvent exposed, we can hypothesize that they may have some impact in forming interactions either with E3 ligases or with other partners. Accordingly, the crystallographic structure of human UBE2B with the E2-binding domain of the cognate E3 ligase Rad18 shows that the differential amino acids are at the interface of the two proteins (Figure 1B).

Available information is in favor of at least partially distinct functions *in vivo* but robust data are clearly needed for clarifying whether close isoforms should be considered as distinct E2 enzymes sharing common properties with their counterparts or whether they are only spare wheels.

## Future directions

This review shows that our knowledge on E2 enzymes in skeletal muscle is very fragmentary, mostly because E3 ligases have been preferentially studied. We now know that E2 enzymes are crucial for the fate of individual substrates and are implicated in major functions like spermatogenesis, mitophagy or the development of pathologies (e.g., cancer). These examples highlight the potential that E2s represent for the future development of therapeutic strategies and underscore the lacks we have in this area. Future studies should decipher the mechanisms involved in substrate ubiquitination in skeletal muscle, keeping in mind that E2-E3-partners-substrates heteromers represent the active entity *in vivo* and that each individual switch within the heteromer may uncover a different mechanism. We can take advantage from other studies to characterize the regulation of muscular enzymes. However, it is hard to predict which of the numerous strategies already described in other cell types/organs will apply for deciphering the precise role of muscle ubiquitinating enzymes and uncovering the contractile proteins they target.

### Conflict of interest statement

The authors declare that the research was conducted in the absence of any commercial or financial relationships that could be construed as a potential conflict of interest.
